# Lord Lister in the City

**Published:** 1907-07-06

**Authors:** 


### Lord Lister in the City.

The Corporation of the City of London has on divers occasions honoured great men by granting them the privileges which belong to the honorary Freemen of the City, but it is questionable whether it has ever acknowledged eminence more worthily or showed its appreciation of genius more happily than when on Friday last it presented that distinction to Lord Lister. The list of honorary Freemen of London is a long one. It includes monarchs and untitled men, but it holds no name that is unworthy to be placed on the roll of the real nobility of the nation. The inclusion in that list of the name of one who has done so much for medical science, and whose work has opened a new era in the history of the art of surgery, reflects as much honour on the Corporation as it does upon Lord Lister.

The presentation was made at the Guildhall by the City Chamberlain, Sir Joseph Dimsdale, who, in presenting Lord Lister with the casket, and in extending to him the right hand of fellowship, briefly and felicitously touched upon the achievements which have made the name of Lister venerated, not only by medical men, but by the laity, who recognise the greatness of the master's work. That name, indeed, as the City Chamberlain rightly said, needs neither embellishment nor the sculptor's art to perpetuate it to posterity. "The work which it has been my great privilege to be engaged in has brought its own all-sufficient reward" was the new Freeman's reply in thanking the Corporation; and everyone must recognise the truth of this statement, made with such modesty and dignity. The time has passed when antisepsis and asepsis were of the nature of party politics in medicine. For a long time the work, that to-day stands beyond the pale of adverse criticism, was neglected, slighted, even scoffed at. To be slighted, misunderstood, wrongly interpreted, and adversely criticised has never yet discouraged the true searcher after truth. More often such stumbling-blocks in the way of universal appreciation and grateful recognition have spurred on the investigators to more earnest and energetic efforts, and in Lord Lister's case they have been productive of the usual results. To-day the work, begun almost half a century ago, which was to leave so enduring an impression upon the science of medicine, has received spontaneous and general recognition. Not only in England, but on the Continent as well, the epoch-making advances in regional surgery and the general progress in the evolution of medicine are considered to be indebted to that work to an extent which only the future historian, adequately and impartially judging the whole, will be able correctly to estimate. To-day, when the carbolic spray is an anachronism and the opponents of Listerism are confined to a small class of selfish men and women, who in their regard for the rights of animals forget the claims of humanity, there is a unanimity of opinion as to the excellence of Lister's work which, to a great man, must indeed be an all-sufficient reward.

The honour which the City of London has done to Lord Lister is in every sense the expression of the nation's sentiment of appreciation for a great service to humanity performed by a great personality. But we do not think that it can, in any sense of the word, be regarded as placing "a coping-stone to the monument" of his fame. The mere granting of civic honours is not an adequate remuneration for a life-work of such brilliancy and merit. We do not doubt that the time will come when the nation, as a whole, will join in expressing its appreciation in a still more tangible form. Lord Lister's work is not merely national; it is not even merely Imperial. It is work that appeals to the whole of humanity without distinction of race or interest. It must indeed be gratifying to find that it is appreciated by the whole world as cordially and as heartily as it has been appreciated by the Corporation of the City of London.

